# Vildagliptin ameliorates biochemical, metabolic and fatty changes associated with non alcoholic fatty liver disease

**DOI:** 10.12669/pjms.326.11133

**Published:** 2016

**Authors:** Mazhar Hussain, Muhammad Zafar Majeed Babar, Muhammad Shahbaz Hussain, Lubna Akhtar

**Affiliations:** 1 Dr. Mazhar Hussain, M.Phil-Pharmacology, Department of Pharmacology, Sheikh Zayed Medical College, Campus Police General Hospital, Jail Chowk, Rahim Yar Khan, Pakistan; 2 Dr. Muhammad Zafar Majeed Babar, FCPS Medicine. Department of Medicine, Medical Unit 1, Sheikh Zayed Medical College & Hospital, Rahim Yar Khan, Pakistan; 3 Dr. Muhammad Shahbaz Hussain, M.Phil- Microbiology. Department of Pathology, Sheikh Zayed Medical College & Hospital, Rahim Yar Khan, Pakistan; 4 Dr. Lubna Akhtar, FCPS Gynae & Obs, Department of Pharmacology, Sheikh Zayed Medical College, Campus Police General Hospital, Jail Chowk, Rahim Yar Khan, Pakistan

**Keywords:** Fatty liver, Vildagliptin, Body weight, Lipid profile, Liver enzymes, Steatosis, Ultrasound, Grading

## Abstract

**Objective:**

To determine the effect of Vildagliptin in non-alcoholic, fatty liver disease patients with dyslipidemia.

**Methods:**

A randomized placebo controlled trial was conducted at outpatient clinic of Medical Unit-I of Sheikh Zayed Medical College/Hospital, Rahim Yar Khan, in which fifty eight patients of NAFLD with dyslipidemia were divided in to two, case and control groups. The case group was given tablet Vildagliptin 50mg twice a day for twelve weeks and control group was given placebo in same way. Body weight, body mass index (BMI), lipid profile, liver enzymes and ultrasound finding of fatty liver were assayed before and after treatment.

**Results:**

After 12 weeks treatment of vildagliptin there was significant improvement in following parameters. Body weight and BMI decreased significantly from 88 ± 11 to79 ± 12 kg (p0.036) and 30±4to 27±5 kg/m^2^ (p 0.005) respectively. Notable reduction in the value of TC, TG and LDL-C (TC:252±24 to 220±20mg/dl (p 0.031); TG: 190±24 to115±22 mg/dl (p 0.005); LDL-C 160±15 to 145±13mg/dl (p 0.004). HDL-C level increased significantly from 29±5to45±4 mg/dl (p 0.001). There was remarkable reduction in aminotransferases level (ALT: 78± 17 to 48±14IU/L (p 0.036). AST: 63.3±13 to41±11IU/L (p 0.002). There was overall 65.5% improvement in fatty liver grading on ultrasound with vildagliptin while non significant effects were seen in placebo group in all of the above parameters.

**Conclusion:**

Vildagliptin exhibited beneficial effects in non-alcoholic fatty liver disease, Non-diabetic patients with dyslipidemia.

## INTRODUCTION

Non alcoholic fatty liver disease (NAFLD) is a global health issue. Its prevalence is much higher in developed countries 20-30% as compared to developing countries 10-20%. However recently it has been documented that prevalence is increasing in developing countries like Pakistan due to epidemics of obesity, metabolic syndrome, diabetes, hypertension and dyslipidemia.^1^

Majority of the patients of NAFLD have no definite signs and symptoms. Most of them are diagnosed on routine medical checkup on the basis of upper vague abdominal discomfort, asymptomatic elevation of serum transaminases and ultrasound finding of fatty liver.^2^ Its natural course progresses from simple benign fatty liver to non alcoholic steatohepatitis (NASH) which leads to fibrosis and then ultimately cirrhosis.^3^ However the leading cause of death in patients of NAFLD is cardiovascular disease and hepato cellular cancer.^4^

NAFLD is diagnosed on the basis of three criteria non alcoholic, fatty liver on ultrasound or histology and absence of other liver disease. Simple fatty steatosis can easily be diagnosed on abdominal ultrasound because it is easily available, cheap, non invasive and provide qualitative information. Other test such as liver biopsy (gold standard), MRI, CT and Fibro scan are valuable in the diagnosis of NAFLD but they have their own hazards such as they are not suitable for population screening on large scale, expensive, require a proper set up with protocol.^5^

The treatment of NAFLD is life style modification and drugs. Life style modification includes weight loss, physical exercise and diet.^6^ There is no licensed therapy for NAFLD up till now; however pharmacological treatment consists of multiple drugs with varying results such as metformin, pioglitazone, antioxidant (vitamin E), silymarin, pentoxifylline and lipid lowering agents such as statins and ezetimibe.^7^

Vildagliptin an oral anidiabetic incretin based therapy used in patients of type 2 diabetes mellitus. It increases the physiological concentration of glucagon like peptide GLP-1 and glucose dependent insulinotropic polypeptide (GIP) in body by inhibiting the enzyme dipeptidyl peptidase –IV (DPP-4) with well tolerated profile, no risk of weight gain and hypoglycemia. Vildagliptin increases GLP level that causes insulin release, inhibits glucagon secretion, delay gastric emptying and reduces appetite.^8^

In addition to blood sugar control vildagliptin has a potential role in the treatment of NAFLD. The proposed mechanism by which vildagliptin produces its beneficial effect in patients of NAFLD include: it improves insulin resistance a key metabolic abnormality in patients of NAFLD.^9^ Second it decreases serum DPP-4 activity because patients with NAFLD have increase DPP-4 which co relates with hepatic steatosis^10^ and finally decrease level of DPP-4 reduces inflammation in patients of NAFLD which is pathognomonic features in these patients.^11^

In addition vildagliptin has beneficial effects on all those risk factors which are associated with NAFLD in various clinical as well as in animal studies such as improvement in metabolic syndrome, blood pressure weight gain and lipid profiles.^12^ Moreover it also has anti-inflammatory and anti oxidant properties because inflammation and oxidative stress plays an important role in the progression as well as complications of NAFLD.^11^

In this study effect of vildagliptin on body weight, lipid profile, hepatic enzyme and sonographic findings of fatty liver has been determined and its independent effect in non diabetic dyslipidemia of non-alcoholic fatty liver disease is seen.

## METHODS

This randomized placebo controlled trial was approved by ethical committee and was conducted at outpatient clinic of Medical Unit-I of Sheikh Zayed Medical College/Hospital, Rahim Yar Khan, from 10 January 2016 to 10 April 2016. Patients presented in medical outdoor with chief complaints of upper abdominal discomfort, dyspepsia and generalized weakness. A written informed consent was obtained from the participants before they were enrolled in the study. Initially one hundred and sixty patients aged 20-65 with BMI ≥ 25 were screened for abnormal liver function test and deranged lipid profile out of which fifty eight patients were enrolled in the study after fulfilling the inclusion and exclusion criteria. The inclusion criteria consisted of presence of fatty liver grading 1, 2, 3 on abdominal ultrasound, mild to moderate elevation of aminotransferases level and deranged lipid profile i.e. cholesterol ≥ 200mg/dl, serum triglycerides≥ 150mg/dl, LDL-Cholesterol ≥ 160mg/dl and HDL-cholesterol ≤ 45mg/dl. All the test and ultrasound were performed at the same centre to avoid discrimination. The exclusion criteria were patients with history of diabetes mellitus, decompensated liver disease, ascities, and esophageal varices and alcohol use. Patients were also screened for HBS Ag, Anti HCV and HIV, hereditary defects of iron, copper and alpha-1 antitrypsin deficiency if they had any history of these conditions in the past, very high level of aminotransferases and highly abnormal ultrasound. Secondary causes of NAFLD such as hypothyroidism, hypogonadism, obstructive sleep apnea, total parenteral nutrition, short bowel syndrome, pancreatodeudenal resection and drugs which cause fatty liver such as corticosteroids, antiviral (nucleoside analogue), tetracycline, methotrexate, tamoxifen and amiodarone were also ruled out. In addition patients who were taking any anti hyperlipidemic and anti diabetic agents were also excluded from the study.

After randomization which was based on random numbers generated by computer, patients were divided in two groups so that effect of vildagliptin can be compared with placebo. First study group was given tablet vildagliptin at a dose of 50mg twice a day while second control group was given tab placebo which was completely similar to the active drug in terms of color, size but it contained sugar as an active ingredient. In both groups treatment were given for a period of 12 weeks. Biochemical, metabolic and fatty changes associated with NAFLD was analyzed before and after the end of study. Fasting blood samples were drawn from the antecubital vein before and at the end of the study. The samples were used for analyzing blood sugar, Lipid profile, AST, ALT and GGT. Fasting blood sugar was measured by glucose oxidize peroxides method to exclude diabetic patients at the start of study. Lipid profile and aminotransferases was done by semi automated clinical chemistry analyzer (Micro lab 300) using spectrophotometer principal.

The high resolution ultrasound machine (Toshiba Xario™ 200) was used to assess fatty liver by experienced radiologist who was unknown to clinical and laboratory data of the study subjects at baseline and after three months treatment with vildagliptin. The classification of NAFLD was based upon the severity of fatty liver on abdominal ultrasound according to the given criteria.^13^

**Grade 0:** No fatty liver

**Grade 1 (Mild):** There was slight diffuse increase in the echogenicity of liver parenchyma or increased hepatorenal contrast with normal diaphragm and intrahepatic vessel borders.

**Grade 2 (Moderate):** There was moderate diffuse increase in the echoegenicity of liver parenchyma and increased hepatorenal contrast with slight impairment of diaphragm and intrahepatic vessel borders.

**Grade 3 (Severe):** In addition to criteria for moderate steatosis there was no visualization of posterior portion of the right lobe of liver, intrahepatic vessel borders and diaphragm.

### Data Analysis

Sample size was calculated to detect difference of over 5 IU/L on transaminases level with 90% power and 5% significance. Statistical package for social sciences SPSS 16 was used for the analysis of data. Values of numeric data were presented as mean ± standard deviation. The differences at baseline between two groups were assessed by t-test. Changes from baseline to 12 weeks were compared by paired t-test within each group and by t-test or Mann-Whitney U-test between groups. Values of p < 0.05 were deemed to be statistically significant.

## RESULTS

All patients completed the study. Both groups tolerated drugs very well with no untoward effects observed during the study period. The baseline demographic characteristics between two groups are shown in Table-I. There were non significant differences between the two groups at baseline in terms of body weight, BMI, lipid profile and aminotransferases level. There were no diabetic patient in this study and this was ruled out by doing fasting blood sugar level at the start of study The fatty liver grading on ultrasound also showed non significant difference (p=0.71). There were no patients in both groups classified in grade zero fatty liver. In Vildagliptin group 24.1% of the patients were classified as grade 1, 58.62% as grade 2 and 17.24% in grade 3 while in placebo group 27.5% patients were classified as 1, 55.17% as grade 2 and 17.24% in grade 3 fatty liver respectively.

**Table-I:** Baseline characteristics of study groups (N-58).

Baseline Characteristics	Vildagliptin (n-29)	Placebo (n-29)	P-value
Age (years)	28±15	31±12	0.74
Sex Male/Female	18/10	20/9	0.88
Body weight (kg)	88±11.3	82 ±16.4	0.03
BMI (Body Mass index kg/m^2^)	30.7±4.2	29.6 ±0.4	0.05
Systolic Blood pressure (mmhg)	120±8.2	115±6.9	0.63
Diastolic Blood pressure (mmhg)	84±8.2	78±9.0	0.67
Blood sugar fasting (mg/dl)	86 ±18.4	83±16.5	0.78
Duration of disease (years)	1.9±3.5	2.2±4.2	0.62
Fatty liver grading (1/2/3)	29(7/17/5)	29(8/16/5)	0.71

Values are given ± standard deviation BMI: body mass index, LDL-Cholesterol: low density lipoprotein cholesterol, HDL-cholesterol: high density lipoprotein cholesterol, ALT: alanine aminotransferases, AST: aspartate aminotransferases, GGT: gamma glutamyl transpeptidase t-test between two groups.

**Table-II:** Results of Vidagliptin and Placebo group (pre and post treatment).

Parameters	Vildagliptin (n-29)		P value*	Placebo (n-29)		P value*	P value^+^

	Pre treatment	Post treatment		Pre-treatment	Post treatment
Body weight(kg)	88±11.3	79±12.6	0.036	82 ±16.4	81±12.4	0.74	0.04
BMI(kg/m^2^)	30.7±4.2	27.5±5.2	0.005	29.6 ±0.4	29.4±0.7	0.88	0.028
TC (mg/dl)	252.6±24.4	220.6±20.2	0.031	260± 28.5	262±28.2	0.62	0.01
TG(mg/dl)	190±24.9	115±22.9	0.005	197±21.2	199±20.4	0.88	0.001
LDL-C(mg/dl	160±15.24	145±13.2	0.004	169±12.8	167±11.2	0.57	0.011
HDL-C(mg/dl)	29.6±5.8	45.5±4.9	0.001	28.3±6.0	33±6.2	0.98	0.02
ALT(IU/L)	78.2±17.2	48.6±14.8	0.036	76±18.9	72±2.2	0.76	0.04
AST(IU/L)	63.5±10.5	41.5± 9.6	0.002	61.2±11.1	58±10.5	0.81	0.001
GGT (IU/L)	18.5±5.8	19.7±7.7	0.62	19.7±6.43	20.8±7.5	0.43	0.67

Results are expressed as mean ± standard deviation.

* P value indicate comparison within groups while

+ P value indicates comparison of changes of each variable between the two groups

BMI: body mass index, TC: total cholesterol, TG: triglycerides, LDL-Cholesterol: low density lipoprotein cholesterol, HDL-cholesterol: high density lipoprotein cholesterol, ALT: alanine aminotransferases, AST: aspartate aminotransferases, GGT: gamma glutamyl transpeptidase Paired t-test within each group and t-test or Mann-Whitney U-test between groups.

After twelve weeks of Vildagliptin therapy body weight decreased significantly from 88±11 to 79±12 kg vs. placebo 88±16 to 81±12kg (p 0.04). Notable reduction in BMI value from 30±4 to 27±5kg/m^2^ vs placebo 29±0.4to 29±0.7 kg/m^2^ (p 0.028). Vildagliptin also causes a remarkable reduction in the value of TC, TG and LDL-C from baseline to end point vs placebo (TC: 252±24 to 220±20mg/dl vs placebo 260± 28to262±28 mg/dl (p0.01); TG: 190±24to 115±22 mg/dl vs. placebo197±21to 199±20mg/dl (p 0.01); LDL-C 160±15 to 145±13mg/dl vs placebo 169±12 to 167±11mg/dl with (p 0.011). Vildagliptin also increased HDL-C level significantly from baseline to 12 weeks vs. placebo from 29±5 to45±4 mg/dl versus placebo 28±6 to 45±4 mg/dl (p 0.02). There is remarkable reduction in aminotransferases level after treatment with vildagliptin versus placebo (ALT: 78± 17 to 48±14 IU/L vs 76±18to 72±2 IU/L (p 0.04). AST:63±13 to41±11 IU/Lvs.61±11 to 58±10 IU/L (p 0.001). There was significant regression in fatty liver grading after 12 weeks treatment with Vildagliptin versus placebo grade 1 fatty liver 100% vs 37.5, grade 2 fatty liver 58.8% vs 12.5%, grade 3 fatty liver 40% vs 20%. Overall 65.5% patients were successful treated in vildagliptin group while in placebo group it was only 20.6% and main impact of vildagliptin treatment at the specific dose was seen in grade 1 -2 fatty liver. These results are shown in Table-III.

**Table-III:** Fatty liver changes in Vidagliptin and placebo treated group (pre & post treatment) N=58.

Vildagliptin group (n= 29)			

Fatty liver grading	Pre treatment	Post treatment	Cases treated
1	7	0	7
2	17	7	10
3	5	3	2

Total	29	10	19

Placebo group (n= 29)			

Fatty liver grading	Pre treatment	Post treatment	Cases treated

1	8	5	3
2	16	14	2
4	5	4	1

Total	29	23	6

## DISCUSSION

In this study vildagliptin causes a significant reduction in body weight after 12 weeks treatment. Most of the patients of NAFLD are obese so body weight reduction via life style modification is the initial step in the management of NAFLD and its effectiveness was proven in various studies.^14^,^15^ Gomez^16^ concluded that more than 10 percent reduction in body weight not only improves NAFLD but also histological features in patients of NASH.

The two most studied anti diabetic agents in patients of NAFLD are metformin and pioglitazone. A meta-analysis of humans and animals studies showed that these drugs are very effective in NAFLD. They act as insulin sensitizers and reduce insulin resistance which is the main metabolic abnormality in NAFLD.^17^ The mechanism by which vildagliptin reduces body weight include its increasing insulin sensitivity in over weight NAFLD patients like metformin. Moreover vildagliptin increases insulin level, decreases glucagon level, decrease appetite and delays gastric emptying which are additional favorable effect in these patients.^18^

A meta-analytic assessment showed that prevalence of hyperlipidemia varies from 60-80% in patients of NAFLD.^19^ In our study all patients were hyperlipidemic it was found that after three months treatment with vildagliptin there was significant improvement in deranged lipid profile especially triglycerides which have strong association with NAFLD. The dyslipidemic effect of vildagliptin may be related to GLP-I mediated decrease in the intestinal lymph flow, inhibition of TG absorption from the intestine and reduced VLDL release from the liver.^20^

In this study vildagliptin caused significant reduction in serum transaminases level. In addition to risk factors discussed above, NAFLD is considered to be an inflammatory disease in which ongoing inflammation and oxidative stress causes elevation of liver enzyme and drugs such as vitamin E and Silymarin which have anti-inflammatory and antioxidant properties are very beneficial in these patients.^21^ The improvement in transaminases level by vildagliptin was related to the reduction of various cytokines and chemokines that are implicated in NAFLD.^22^ Most of the clinical and animals studies of DPP-4 inhibitors were on diabetes in which vildagliptin improved NAFLD and hepatic steatosis by reducing triglycerides, aminotransferases and inflammation.^23^-^25^ In addition sitagliptin which is another DPP-4 inhibitor ameliorates hepatic ballooning and steatosis score in NASH patients with type 2 diabetes when given for a period of one year. NASH is the advanced stage of NAFLD and may progress to cirrhosis and even cancer.^26^

While in non diabetic patients the most beneficial drugs are vitamin E, pioglitazone and silymarin and their effects was proven in various clinical trials.^21^,^27^-^28^ However still so far no data is available to see the effect of vildagliptin in NAFLD in non diabetics. In our study which was conducted on non diabetic patients vildagliptin not only reduced body weight, lipid profile and serum transaminases level but also showed more than 60% of regression of fatty liver on ultrasound in the form of decreased liver brightness and hepatorenal contrast after three months treatment. Although ultrasound is not a gold standard for NAFLD but its sensitivity is between 60-90% and it is comparable with MRI, CT- scan and liver biopsy.^29^

## CONCLUSION

Vildagliptin exhibited beneficial effects in non-alcoholic fatty liver disease, Non-diabetic patients with dyslipidemia.

## RECOMMENDATION

There is high plasma DPP-4 activity in patients of NAFLD who are either diabetic or non diabetic. Early use of vildagliptin should be recommended in these patients in order to prevent its ongoing complications because Vildagliptin decreases plasma DPP-4 which is positively correlated with liver enzymes and considered to be novel disease biomarker in future.^30^,^31^ Future studies with large sample size and of longer duration should be conducted.

### Limitations of the Study

There was limited number of patients in grade 3 fatty liver which responded less as compared to study to grade 1-2 fatty liver, it may be due to short duration of study period and reduced sample size. In addition lack of histological finding by liver biopsies (gold standard) was also a limitation of this study but it was not performed due to its invasiveness and low acceptance rate in patients of simple NAFLD.
